# How to distinguish crowned dens syndrome from acute meningitis?

**DOI:** 10.1002/jgf2.644

**Published:** 2023-10-06

**Authors:** Saori Adachi, Toshiki Uchihara, Shuta Toru

**Affiliations:** ^1^ Department of Neurology Nitobe Memorial Nakano General Hospital Nakano Japan

To the Editor,

We read the article by Isono et al.^1^ with great interest. We were impressed not only by the largest number of cases recruited into this collaborative study but also by the detailed data collection under a unified protocol, which successfully delineated clinical features of crowned dens syndrome (CDS).

As clarified, neck pain exacerbated by rotation or pressure, accompanied by fever and an elevated inflammatory response on blood tests strongly suggests CDS. However, this clinical picture may mimic acute meningitis with head/neck pain with jolt accentuation especially when accompanied by fever.^2^ For example, one of our patients presented with neck pain accompanied by headache, without fever or loss of consciousness, but an increased cell count on CSF led to the diagnosis of aseptic meningitis. Therefore, neck pain does not preclude the possibility of meningitis. Furthermore, Mizumoto^3^ reported a CDS patient with pleocytosis, which alert the possibility of meningitis even in the presence of CDS. Because it is not yet known when calcification around the dens as a hallmark of CDS appears, persists, or disappears, overreliance on calcification around dens may overlook CDS mimics like meningitis. Indeed, among the CDS patients by Isono and colleagues, 8% had impaired consciousness and 15% underwent lumbar puncture, suggesting that some of these CDS patients may mimic meningitis to warrant an emergent need of lumbar puncture. Although our department of neurology shares this feeling, the major difficulty is how to select candidates for lumbar puncture to rule out possible coexistence of meningitis. It is necessary to establish clinical criteria for CDS in distinction from meningitis, which needs a prospective approach. Before such criteria are available, indication of lumbar puncture may remain relative. Therefore, it is currently very informative if the authors are ready to disclose how the indication of lumbar puncture was decided during this study. This is necessary and helpful to establish reliable management of CDS.

## CONFLICT OF INTEREST STATEMENT

The authors declare no conflict of interest regarding this submission.
